# Associations between quantitative [^18^F]flortaucipir tau PET and atrophy across the Alzheimer’s disease spectrum

**DOI:** 10.1186/s13195-019-0510-3

**Published:** 2019-07-04

**Authors:** Tessa Timmers, Rik Ossenkoppele, Emma E. Wolters, Sander C. J. Verfaillie, Denise Visser, Sandeep S. V. Golla, Frederik Barkhof, Philip Scheltens, Ronald Boellaard, Wiesje M. van der Flier, Bart N. M. van Berckel

**Affiliations:** 1 Department of Radiology, Amsterdam UMC, location VUmc, P.O. Box 7057, 1007 MB Amsterdam, The Netherlands; 2 Amsterdam Alzheimer Center, Amsterdam UMC, location VUmc, P.O. Box 7057, 1007 MB Amsterdam, The Netherlands; 3 Institutes of Neurology and Healthcare Engineering, UCLK, London, UK; 4 Department of Epidemiology and Biostatistics, Amsterdam UMC, location VUmc, Amsterdam, The Netherlands; 5 Clinical Memory Research Unit, Lund University, Lund, Sweden

**Keywords:** Tau, Atrophy, Alzheimer’s disease, MCI (mild cognitive impairment), MRI, PET

## Abstract

**Background:**

Neuropathological studies have linked tau aggregates to neuronal loss. To describe the spatial distribution of neurofibrillary tangle pathology in post-mortem tissue, Braak staging has been used. The aim of this study was to examine in vivo associations between tau pathology, quantified with [^18^F]flortaucipir PET in regions corresponding to Braak stages, and atrophy across the Alzheimer’s disease (AD) spectrum.

**Methods:**

We included 100 subjects, including 58 amyloid-β positive patients with mild cognitive impairment (MCI, *n* = 6) or AD dementia (*n* = 52) and 42 controls with subjective cognitive decline (36% amyloid-β positive). All subjects underwent a dynamic [^18^F]flortaucipir PET to generate non-displaceable binding potential (BP_ND_) maps. We extracted average [^18^F]flortaucipir BP_ND_ entorhinal, Braak III–IV (limbic) and Braak V–VI (neocortical) regions of interest (ROIs). T1-weighted MRI was used to assess gray matter (GM) volumes. We performed linear regression analyses using [^18^F]flortaucipir BP_ND_ ROIs as independent and GM density (ROI or voxelwise) as dependent variable.

**Results:**

In MCI/AD subjects (age [mean ± SD] 65 ± 8 years, MMSE 23 ± 4), [^18^F]flortaucipir BP_ND_ was higher than in controls (age 65 ± 8, MMSE 29 ± 1) across all ROIs (entorhinal 0.06 ± 0.21 vs 0.46 ± 0.25 *p* < 0.001, Braak III–IV 0.11 ± 0.10 vs 0.46 ± 0.26, *p* < 0.001, Braak V–VI 0.07 ± 0.07 vs 0.38 ± 0.29, *p* < 0.001). In MCI/AD, greater [^18^F]flortaucipir BP_ND_ in entorhinal cortex was associated with lower GM density in medial temporal lobe (*β* − 0.40, *p* < 0.001). Greater [^18^F]flortaucipir BP_ND_ in ROI Braak III–IV and Braak V–VI was associated with smaller GM density in lateral and inferior temporal, parietal, occipital, and frontal lobes (range standardized *β*s − 0.30 to − 0.55, *p* < 0.01), but not in medial temporal lobe (*β* − 0.22, *p* 0.07). [^18^F]Flortaucipir BP_ND_ in ROI Braak I–II was not associated with GM density loss anywhere. When quantifying [^18^F]flortaucipir BP_ND_ across brain lobes, we observed both local and distant associations with GM atrophy. In controls, there were no significant associations between [^18^F]flortaucipir BP_ND_ and GM density (standardized *β*s ranging from − 0.24 to 0.02, all *p* > 0.05).

**Conclusions:**

In MCI/AD patients, [^18^F]flortaucipir binding in entorhinal, limbic, and neocortical regions was associated with cortical atrophy.

**Electronic supplementary material:**

The online version of this article (10.1186/s13195-019-0510-3) contains supplementary material, which is available to authorized users.

## Introduction

Accumulation of tau and amyloid-beta (Aβ) proteins are the pathological hallmarks of Alzheimer’s disease (AD) [1, 2]. Post-mortem studies suggest that tau spreads in a stereotypical pattern during the disease course, which can be captured by the Braak staging system of neurofibrillary tangle pathology [3, 4]. In this staging scheme, AD-related tau pathology is restricted to the (trans) entorhinal cortex in Braak stages I–II, spreads through medial and inferior temporal lobes in stages III–IV, and finally involves isocortical brain areas in stages V–VI.

Brain atrophy in AD is the downstream result of a cascade of poorly understood processes in which tau pathology may play an important role [5, 6]. Atrophy in AD typically affects the entorhinal cortex and hippocampus [7, 8], but several other vulnerable regions have been identified, including the temporal pole, inferior parietal lobe and posterior cingulate cortex [9]. Neuropathological studies in AD have linked hyperphosphorylated tau aggregates to neuronal loss [10, 11]. However, neuropathological studies are limited by the long interval between autopsy and symptom onset, and histopathological evaluation does not provide full brain coverage. Positron emission tomography (PET) and the tau tracer [^18^F]flortaucipir now allow whole-brain examination of tau pathology in vivo. Previous studies have shown that [^18^F]flortaucipir retention patterns mirror the Braak stages [12–15] further validating [^18^F]flortaucipir PET as an in vivo marker for hyperphosphorylated tau.

First reports evaluating the associations between tau PET and markers for neurodegeneration showed strong associations between tau burden and local neurodegeneration, predominantly in temporal regions [16–26]. However, most studies included subjects ranging from cognitively unimpaired to patients with AD dementia, while studies restricting analyses to patients with a clinical diagnosis of AD dementia are sparse and have relatively small sample sizes [16, 20, 23, 26]. The strength of relationships between tau and neurodegeneration seem to vary by brain region, and tau in a given region can be related to neurodegeneration in a spatially distant region. In cognitively normal participants, associations between tau and distant atrophy or FDG metabolism revealed complex patterns, dependent on amyloid-β and the tau region under investigation [18, 21, 22]. While some studies reported tau-associated neurodegeneration in adjacent regions only [16, 17], others reported widespread distant atrophy [18, 19].

Here we investigated the associations between tau pathology and MRI measures of atrophy in a memory clinic population of AD dementia, mild cognitive impairment (MCI), and cognitively unimpaired participants with subjective cognitive decline (SCD). We used dynamic [^18^F]flortaucipir PET to accurately quantify tau load and investigated the local and distant associations between tau PET and gray matter density. To capture the spatial distribution of tau, we examined tau in three composite regions reflecting Braak I–II, III–IV, and V–VI stages. In addition, for a more unbiased approach, we quantified tau in multiple regions of interest across brain lobes, repeated analyses per clinical subgroup, and assessed both local and distant associations between tau and atrophy.

## Materials and methods

### Subjects

We included 100 subjects from the Amsterdam Dementia Cohort with [^18^F]flortaucipir PET and structural MRI scans. All subjects visited the memory clinic of the Alzheimer Center Amsterdam for a standardized dementia screening, including medical and neurological examination, informant-based history, assessment of vital functions, screening laboratory tests, neuropsychological evaluation, MRI, and lumbar puncture and/or amyloid-β PET. Diagnoses were determined in a multidisciplinary consensus meeting [27, 28]. We included subjects with a diagnosis of Alzheimer’s disease dementia [29], mild cognitive impairment (MCI) due to Alzheimer’s disease [30], and cognitively normal controls with subjective cognitive decline (SCD) [31]. Participants with SCD had cognitive complaints, but cognitive functioning was within normal limits and there was no other neurological or psychiatric disorder present. For all subjects, AD biomarkers in the cerebrospinal fluid (CSF) and/or Aβ PET were available. All MCI and dementia subjects had abnormal AD biomarkers (CSF Aβ_42_ < 813 pg/mL [32] and/or abnormal Aβ PET ([^11^C]PiB (*n* = 2), [^18^F]florbetaben (*n* = 24), or [^18^F]flutemetamol (*n* = 2) on visual read)) [33]. MCI and AD dementia subjects were included based on the presence of abnormal Aβ biomarkers. In the case of conflicting AD biomarkers, one abnormal biomarker (PET or CSF) was considered sufficient for inclusion. All controls participated in a longitudinal study on SCD (the SCIENCe cohort) [28]. All participants underwent [^18^F]flortaucipir PET and MRI scans, with a time interval between PET and MRI of maximum 6 months (MCI and AD) or 12 months (controls). Exclusion criteria were severe traumatic brain injury, abnormalities on MRI likely to interfere with segmentation of tau PET, and participation in drug trial with a tau or Aβ-targeting agent. All participants gave written informed consent to participate. The Medical Ethics Review Committee of the VU University Medical Center approved this study.

### Image acquisition

All structural whole-brain MRI scans were performed on a single 3.0-T Philips Ingenuity Time-of-Flight PET/MR scanner (Philips Medical Systems, Best, The Netherlands). Isotropic structural 3D T1-weighted images were acquired using a sagittal turbo gradient-echo sequence (1.00 mm^3^ isotropic voxels, repetition time = 7.9 ms, echo time = 4.5 ms, and flip angle = 8°).

Tau PET images were acquired on a PET/CT scanner (Ingenuity TF [*n* = 94], Gemini TF-64 [*n* = 6], both Philips Medical Systems, Best, The Netherlands). Individual doses of [^18^F]flortaucipir were prepared on site in accordance with Avid Radiopharmaceuticals quality control release criteria [34]. Head movements were restricted by using a headband. During scan procedures, head movement was monitored using laser beams and, if necessary, corrected. After a low-dose CT for attenuation correction, 237 ± 14 MBq [^18^F]flortaucipir was injected (injected mass 1.15 ± 0.85 μg) simultaneously with the start of a 60-min dynamic emission scan. After a 20-min break and a second low-dose CT, a second dynamic emission scan was started from 80 to 130 min post injection. List mode data were reconstructed using 3D RAMLA with a matrix size of 128 × 128 × 90 and a voxel size of 2 × 2 × 2 mm^3^. The second scan session was co-registered to the first, and the two parts were combined into a single data set of 29 frames using an in-house developed code consisting of a multi-frame co-registration feature of Vinci implemented in IDL. Standard corrections (using Philips Healthcare software) for dead time, decay, attenuation, randoms, and scatter were performed [34].

### PET image analysis

T1-weighted MR images were co-registered to their individual PET scans in native space using Vinci software (Max Plank Institute, Cologne, Germany). The Hammers template [35], incorporated in PVElab software, was used to delineate cerebellar gray matter region of interest (ROI) on the co-registered MR images. Receptor parametric mapping (RPM) [36] with cerebellar gray matter as a reference region was applied to the dynamic 130-min PET data, to generate voxelwise parametric images of non-displaceable binding potential (BP_ND_). BP_ND_ is a measure of specific binding, as it reflects the concentration of specifically bound tracer relative to the concentration of free and non-specifically bound tracer in tissue under equilibrium [37]. In previous work, our group reported that RPM is the most optimal parametric method for [^18^F]flortaucipir, when compared with full kinetic modeling [38]. Furthermore, in previous work, no differences in cerebellar gray matter distribution volume (*V*_T_) between controls and AD patients were observed, suggesting cerebellar gray matter is a suitable reference region [34].

We performed partial volume correction (PVC) using Van Cittert iterative deconvolution methods (IDM), a method that improves spatial resolution without the need of structural MRI, combined with highly constrained back-projection (HYPR), to decrease noise [39, 40]. This previously validated method has been shown to improve accuracy in dynamic brain PET in controls and AD patients [41]. All analyses were performed with and without PVC.

We delineated regions of interest (ROIs) on MRI using two different approaches. First, we used four a priori defined non-overlapping ROIs, corresponding to Braak staging regions of tau pathology [3], partially based on Schöll et al. [12]. For these pathology-based ROIs, we created two regions reflecting early tau pathology: an entorhinal and a hippocampal ROI (separated because the hippocampal signal is potentially affected by spill-in from neighboring choroid plexus binding). Furthermore, we combined parahippocampal, fusiform, lingual gyrus, amygdala, inferior temporal, middle temporal cortex, temporal pole, thalamus, caudal, rostral, isthmus, posterior cingulate and insula for Braak III–IV and frontal, parietal, occipital cortex; transverse, superior temporal cortex, precuneus, banks of superior temporal sulcus, nucleus accumbens, caudate nucleus, putamen, precentral gyrus, postcentral gyrus, paracentral gyrus, cuneus, and pericalcarine for Braak V–VI. Next to these pathology-based ROIs, for our second approach, we aimed to quantify tau independent of Braak staging, with a more unbiased method. Therefore, we created seven pre-defined cortical ROIs, covering all major brain lobes. For this purpose, we used the automatic anatomic labeling (AAL) atlas and created ROIs corresponding to the hippocampus, medial temporal (hippocampus, parahippocampal gyrus, amygdala), lateral temporal (inferior temporal gyrus, middle temporal gyrus, superior temporal gyrus), medial parietal (posterior cingulum, precuneus), lateral parietal (inferior parietal gyrus, superior parietal gyrus, supramarginal gyrus), occipital (inferior occipital gyrus, middle occipital gyrus, superior occipital gyrus), frontal (middle frontal gyrus, and superior frontal gyrus) lobes. In addition, we created a global cortical ROI, including all cortical AAL regions.

All co-registered T1-weighted MRI scans were warped to Montreal Neurological Institute (MNI152) space, and these transformation matrixes were used to warp native space BP_ND_ images to MNI space. All warped images were checked manually for transformation errors. We transformed to template space because the Braak masks provided to us were already in template space. For uniformity, we extracted the other ROIs in template space too. Since the coordinates of our masks were slightly different from our MNI-transformed [^18^F]flortaucipir images, we next resliced the transformed BP_ND_ images to AAL (for lobar ROIs) or FreeSurfer (for Braak ROIs) coordinates. For each subject, we obtained volume-weighted BP_ND_ values for each ROI using the MarsBar package implemented within SPM12.

### MR image analysis

We calculated gray matter (GM) density using voxel-based morphometry (VBM) procedures implemented in Statistical Parametric Mapping (SPM) version 12 software (Wellcome Trust Center for Neuroimaging, University College London, UK). First, structural T1-weighted MR images were segmented into the gray matter, white matter, and cerebrospinal fluid (CSF). All gray matter native space images were manually checked for segmentation errors. Next, gray matter segmentations were warped to MNI152 space, using a study specific template created with the DARTEL toolbox [42]. Finally, whole-brain gray matter maps were smoothed with an 8-mm full-width at half-maximum Gaussian kernel. The resulting maps were used for voxelwise analyses. For ROI-based analyses, we extracted regional gray matter density, using the same 7 cortical ROIs as for PET analyses, based on the AAL atlas. In addition, we extracted gray matter density in entorhinal cortex, based on the Freesurfer atlas (since this region is not separately available in AAL atlas). Native space segmentations (gray matter, white matter, and CSF) were combined to calculate total intracranial volume (TIV) for each subject, which was included in all analyses as a nuisance variable. We used GM density as a proxy for atrophy.

### Statistical analysis

In our first approach, we aimed to assess the relationships between tau in regions corresponding to the Braak neuropathological staging scheme and gray matter density. We performed linear regression models with [^18^F]flortaucipir BP_ND_ within each pathology-based region as an independent variable and regional gray matter density as a dependent variable. In order to validate the results obtained when quantifying tau in pathology-based regions, we repeated these analyses with [^18^F]flortaucipir BP_ND_ within each lobar ROI as an independent variable. In addition to the regional analyses, we performed voxelwise analyses with [^18^F]flortaucipir BP_ND_ within each pathology-based region as an independent variable and whole-brain voxelwise gray matter density maps as a dependent variable. We applied both a conservative (*p* < 0.05 familywise error (FWE) corrected) and a more liberal (*p* < 0.001 uncorrected for multiple comparisons) threshold, while adjusting for age, sex, TIV, and PET scanner type. We report results from analyses using partial volume effect (PVE)-corrected [^18^F]flortaucipir images in the main text and from non-PVE-corrected analyses in the Additional files. In our second approach, we used lobar ROIs to assess relationships between [^18^F]flortaucipir BP_ND_ and GM in two ways: (I) local, i.e., between [^18^F]flortaucipir BP_ND_ and GM in the same ROI, and (II) spatially distant, i.e., [^18^F]flortaucipir BP_ND_ in one ROI and GM density in all other ROIs. All analyses were adjusted for age, sex, TIV, and PET scanner type. We reported standardized *β*s and considered a *p* value < 0.05 as significant. We indicated the level of significance both without and with correction for multiple comparisons using the Benjamini-Hochberg procedure with a false discovery rate (FDR) *Q* value of 5%. All analyses were first performed on the total sample and then separately for controls and for MCI/AD patients.

## Results

### Demographics

Demographics according to the diagnostic group are shown in Table 1. Our sample was relatively young, with a mean age of 65 ± 8 years. As expected, subjects in the MCI/AD group had lower scores on MMSE (22.5 ± 3.8 for MCI/AD vs 28.7 ± 1.3 for controls). By definition, all MCI/AD subjects had abnormal amyloid biomarkers, while 36% of the controls had abnormal amyloid biomarkers.

**Table 1:** Demographics

	Total group *n* = 100	Controls *n* = 42	MCI/AD *n* = 58
Age	65 ± 7.6	65.4 ± 7.7	64.7 ± 7.6
Gender (M/F)	55/45	22/20	33/25
MMSE	25.1 ± 4.2	28.7 ± 1.3	22.5 ± 3.8
Aβ positive (%)	73%	36%	100%
Time lag between PET and MRI (months)	3.5 ± 3.4	5.7 ± 3.6	1.8 ± 1.9
TIV	1.4 ± 0.1	1.4 ± 0.1	1.4 ± 0.1

### Regional values for [^18^F]flortaucipir BP_ND_ and gray matter density

**Fig. 1:**
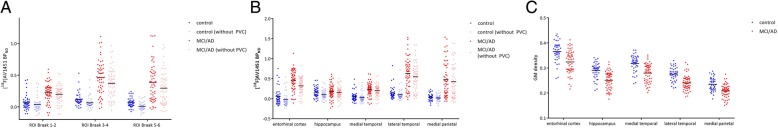
Spatial distribution of [^18^F]flortaucipir BP_ND_ and region GM density. **a** [^18^F]Flortaucipir BP_ND_ per entorhinal, Braak III/IV (limbic), and Braak V/VI (neocortical) ROI. **b** [^18^F]Flortaucipir BP_ND_ for five lobar ROIs. **c** GM density, as extracted from GM density maps, corrected for TIV for five ROIs. For [^18^F]flortaucipir, data are PVE corrected (dark colors) and non-PVE corrected (light colors)

Figure 1 shows the [^18^F]flortaucipir BP_ND_ values (both with and without PVC), for Braak ROIs (a) and lobar ROIs (b). The MCI/AD group had higher mean [^18^F]flortaucipir BP_ND_ in each pathology-based ROI (entorhinal 0.06 ± 0.21 vs 0.46 ± 0.25 *p* < 0.001, Braak III–IV 0.11 ± 0.10 vs 0.46 ± 0.26, *p* < 0.001, Braak V–VI 0.07 ± 0.07 vs 0.38 ± 0.29, *p* < 0.001) than the control group. For lobar ROIs, MCI/AD subjects had higher [^18^F]flortaucipir BP_ND_ in all regions than controls, except for the hippocampus (0.16 ± 0.13 vs 0.18 ± 0.18, *p* = 0.67) (Additional file 7: Table S3). Differences between data with and without PVC were small, ranging from 0.07 ± 0.04 BP_ND_ for Braak III–IV to 0.30 ± 0.08 BP_ND_ for the entorhinal cortex. Regional gray matter density for the entorhinal cortex, hippocampus, medial and lateral temporal, and medial parietal lobes, corrected for TIV, are shown in Fig. 1c. All regional gray matter density was lower for the control group than for MCI/AD.

### Region-of-interest-based correlations

**Fig. 2:**
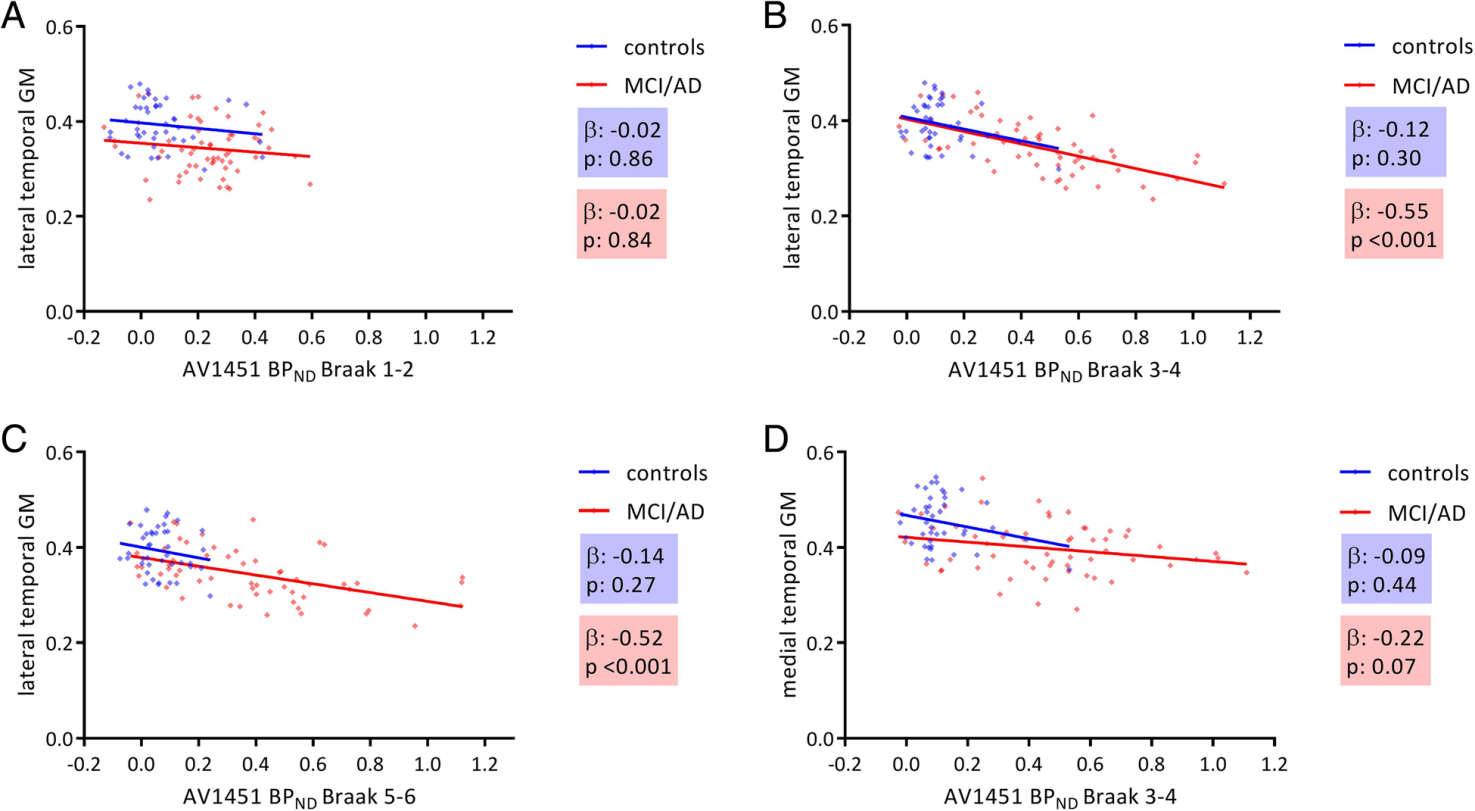
Associations between [^18^F]flortaucipir BP_ND_ and GM density in (**a**) Braak 1-2 and lateral temporal lobe, (**b**) Braak 3-4 and lateral temporal lobe, (**c**) Braak 5-6 and lateral temporal lobe, and (**d**) Braak 3-4 and medial temporal lobe>

Table 2 and Fig. 2 show the associations between [^18^F]flortaucipir BP_ND_ within each pathology-based region of interest and gray matter density in the total sample, as well as stratified by clinical syndrome for controls and the MCI/AD groups separately. Across all subjects, higher [^18^F]flortaucipir BP_ND_ in each pathology-based ROI was correlated to lower GM density (*β*s ranging from − 0.22 to − 0.63, all *p* < 0.01_FDR_). When we stratified for clinical syndrome, we found that in MCI/AD, higher [^18^F]flortaucipir BP_ND_ in the entorhinal cortex was associated with lower GM density in the entorhinal cortex, hippocampus, and medial temporal lobe (*β*s ranging from − 0.38 to − 0.42, all *p* < 0.01_FDR_). Higher [^18^F]flortaucipir BP_ND_ in Braak III–IV ROI was associated with lower GM density in lateral temporal, medial parietal, lateral parietal, occipital, and frontal lobes and the global cortical region (*β*s ranging from − 0.30 to − 0.55, all *p* < 0.01_FDR_), but not significantly with gray matter density reduction in medial temporal lobes (*β* − 0.22, *p* = 0.07) (Fig. 2b, d) anymore. In addition, higher [^18^F]flortaucipir BP_ND_ in Braak V–VI ROI was associated with lower gray matter density in lateral temporal (Fig. 2c), medial parietal, lateral parietal, occipital, and frontal lobes and the global region (*β*s ranging from − 0.33 to − 0.53, all *p* < 0.01_FDR_). In the control group, we found no associations between [^18^F]flortaucipir BP_ND_ and gray matter density (Table 2, Additional file 1: Figure S1). We observed similar results when using non-PVE-corrected [^18^F]flortaucipir BP_ND_ values (Additional file 7: Table S3).

**Table 2:** Correlations between [^18^F]flortaucipir BP_ND_ and regional GM density in the total group, cognitively normal individuals and MCI/AD

	Entorhinal cortex	Hippocampus	Medial temporal	Lateral temporal	Medial parietal	Lateral parietal	Occipital	Frontal	Global
Total group									
[^18^F]Flortaucipir BP_ND_ entorhinal cortex	− 0.422**#	− 0.505**#	− 0.491**#	− 0.424**#	− 0.362**#	− 0.358**#	− 0.392**#	− 0.335**#	− 0.377**#
[^18^F]Flortaucipir BP_ND_ in ROI Braak III–IV	− 0.410**#	− 0.439**#	− 0.439**#	− 0.598**#	− 0.493**#	− 0.475**#	− 0.552**#	− 0.378**#	− 0.472**#
[^18^F]Flortaucipir BP_ND_ in ROI Braak V–VI	− 0.344**#	− 0.393**#	− 0.385**#	− 0.592**#	− 0.548**#	− 0.546**#	− 0.627**#	− 0.405**#	− 0.499**#
Cognitively normal									
[^18^F]Flortaucipir BP_ND_ entorhinal cortex	0.004	− 0.090	− 0.080	− 0.087	− 0.116	− 0.027	− 0.129	− 0.198	− 0.114
[^18^F]Flortaucipir BP_ND_ in ROI Braak III–IV	− 0.087	− 0.070	− 0.089	− 0.120	− 0.190	− 0.048	− 0.015	− 0.033	− 0.079
[^18^F]Flortaucipir BP_ND_ in ROI Braak V–VI	− 0.057	− 0.053	− 0.068	− 0.137	− 0.229	− 0.122	− 0.064	− 0.130	− 0.099
MCI/AD									
[^18^F]Flortaucipir BP_ND_ entorhinal cortex	− 0.378**#	− 0.422**#	− 0.404**#	− 0.226*	− 0.116	− 0.132	− 0.114	− 0.210*	− 0.211*
[^18^F]Flortaucipir BP_ND_ in ROI Braak III–IV	− 0.256*	− 0.209	− 0.224	− 0.555**#	− 0.350**#	− 0.348**#	− 0.432**#	− 0.299**#	− 0.393**#
[^18^F]Flortaucipir BP_ND_ in ROI Braak V–VI	− 0.132	− 0.119	− 0.123	− 0.529**#	− 0.451**#	− 0.460**#	− 0.548**#	− 0.329**#	− 0.431**#

*p < 0.05, **p < 0.01, #FDR corrected

**Fig. 3:**
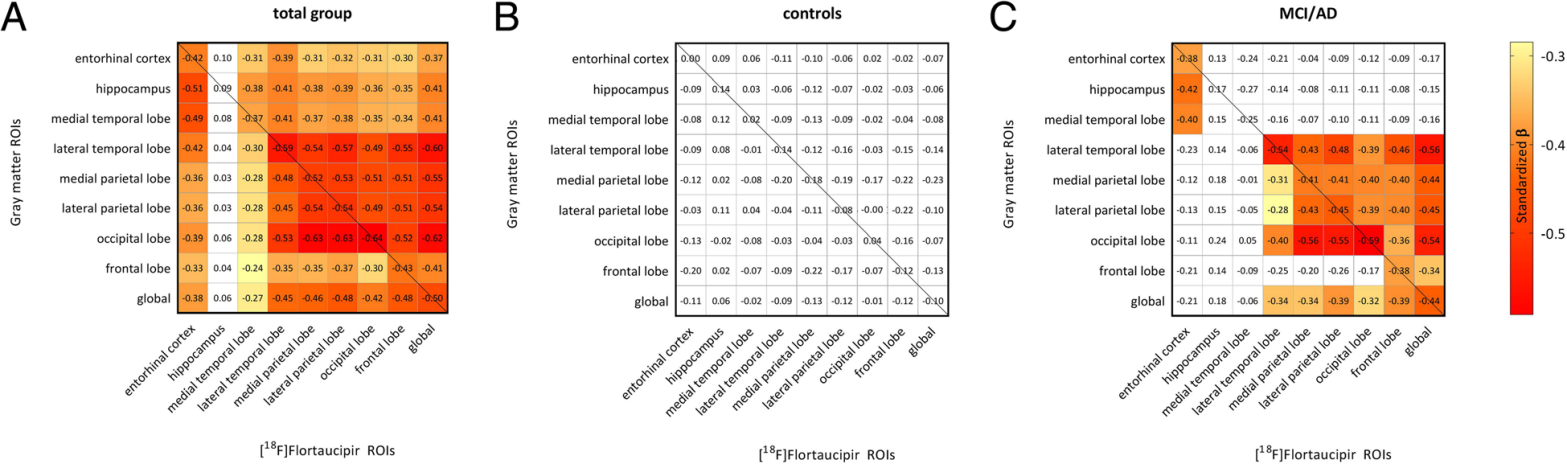
Correlation matrix between regional [^18^F]flortaucipir and GM density for **a** the total sample, **b** controls, and **c** MCI/AD. Analyses are adjusted for age, sex, TIV, and PET scanner type. Displayed are standardized *β*s. Colored boxes indicate a significant association (*p* < 0.05), while white boxes indicate a non-significant association. The color scale represents the strength of the standardized *β*s

Figure 3 shows the correlations between [^18^F]flortaucipir BP_ND_ within each lobar region and regional gray matter density for the total sample (Fig. 3a), and for controls and MCI/AD separately (Fig. 3b, c). The diagonal correlations (line) indicate local associations between [^18^F]flortaucipir BP_ND_ and GM density, whereas off-diagonal elements represent more distant effects (i.e., GM density changes in regions not similar to the tau ROI). Additional file 6: Table S2 shows the significance levels. In the total sample, higher [^18^F]flortaucipir BP_ND_ was correlated to both local and distant GM density reduction. These relationships were present for all lobar regions, except for the hippocampus. When we restricted analyses to diagnostic subgroups, amongst MCI/AD subjects, we again observed local associations between [^18^F]flortaucipir BP_ND_ and GM density reduction in all ROIs except the hippocampus. Distant associations between [^18^F]flortaucipir BP_ND_ and lower GM density were present in the lateral temporal, parietal, occipital, and frontal lobes (*β*s ranging from − 0.25 to − 0.59 all *p* < 0.05). [^18^F]Flortaucipir BP_ND_ in the medial temporal lobe was correlated with smaller GM density in the hippocampus and medial temporal lobe only, but this did not survive correction for multiple comparisons. Furthermore, [^18^F]flortaucipir BP_ND_ had no distant associations with smaller GM density in the hippocampus and medial temporal lobe. We observed no correlations between [^18^F]flortaucipir BP_ND_ and GM density in the control group. When repeating the analyses using non-PVE-corrected data, we found largely similar results (Additional file 7: Table S3).

### Voxelwise correlations

**Fig. 4:**
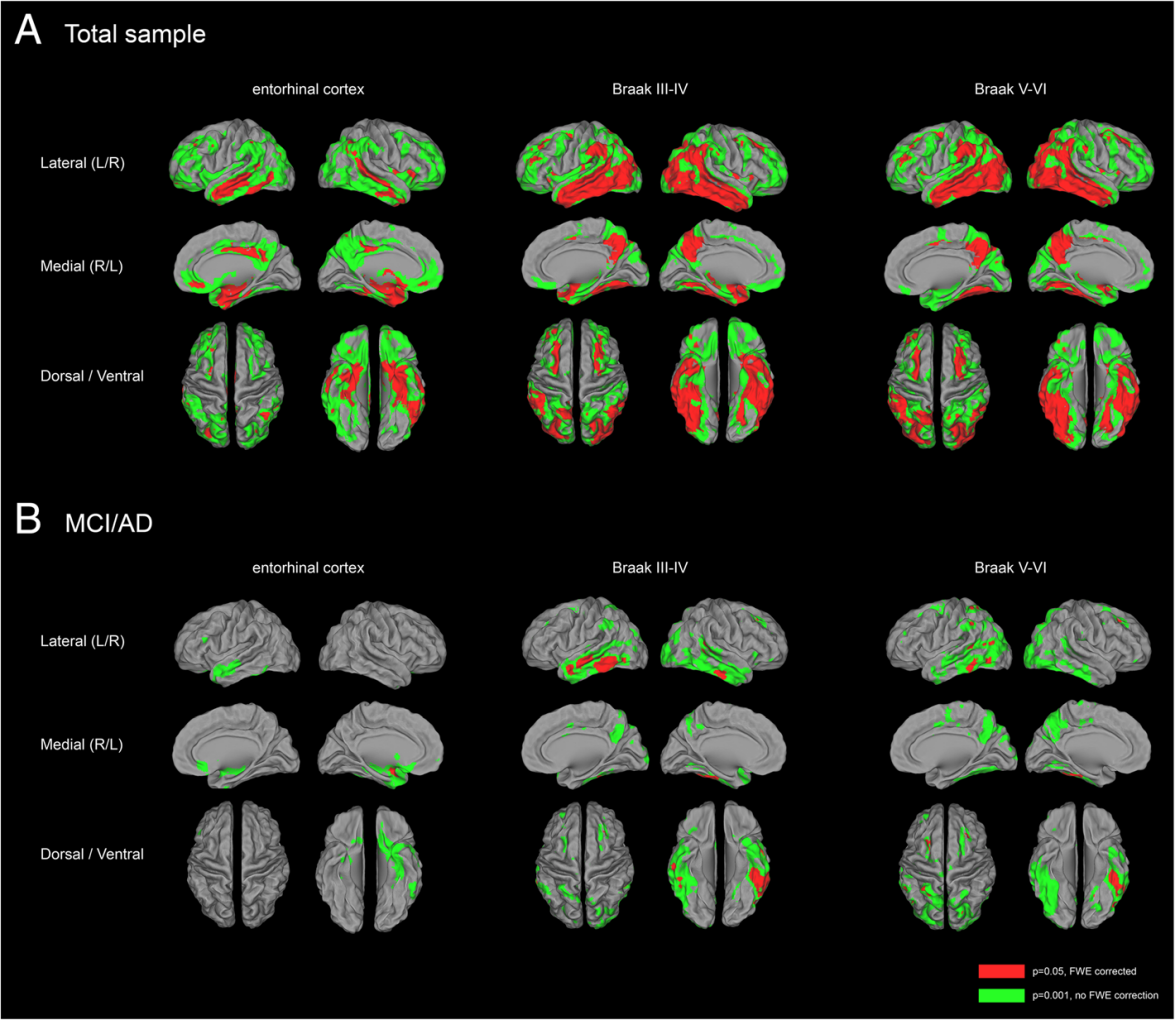
Correlations between [^18^F]flortaucipir BP_ND_ and voxelwise GM density. Displayed are the results of voxelwise regression analyses with [^18^F]flortaucipir BP_ND_ per pathology-based ROI (top) as predictor and GM density as a dependent variable for **a** the total sample and **b** MCI/AD. Analyses are adjusted for age, sex, TIV, and PET scanner type. [^18^F]Flortaucipir BP_ND_ images are partial volume corrected. *P* values are set at 0.05, family-wise error corrected (red), and with a more liberal threshold of *p* < 0.001 (uncorrected for multiple comparisons)

Voxelwise analyses confirmed associations between [^18^F]flortaucipir BP_ND_ in all Braak ROIs and gray matter density reductions in the total sample (Additional file 5: Table S1). Figure 4a shows the correlations of [^18^F]flortaucipir BP_ND_ in the 3 ROIs with voxelwise cortical gray matter density. Higher [^18^F]flortaucipir BP_ND_ in the entorhinal cortex was correlated with a gray matter density pattern that was less widespread than the pattern associated with [^18^F]flortaucipir BP_ND_ in the other Braak ROIs. When we repeated analyses stratified by clinical stage, voxelwise analyses confirmed the absence of any association between [^18^F]flortaucipir BP_ND_ and gray matter density in controls (Additional file 2: Figure S2), and the presence of a negative correlation between [^18^F]flortaucipir BP_ND_ in the entorhinal cortex, Braak III–IV and Braak V–VI, and gray matter density in the MCI/AD group (Fig. 4b, Additional file 3: Figure S3). When applying a threshold of *p* < 0.05_FWE_, higher [^18^F]flortaucipir BP_ND_ in the entorhinal cortex was associated with lower gray matter density in a small region in the left middle temporal lobe. [^18^F]Flortaucipir BP_ND_ in Braak III–IV was associated with a gray matter density reduction pattern affecting the middle and inferior temporal lobes, with a left hemisphere predominance. The gray matter density pattern associated with [^18^F]flortaucipir BP_ND_ in Braak V–VI was comparable to that observed in Braak III–IV, although less pronounced in the left middle and inferior temporal lobes and non-significant in the right temporal lobe. In addition, we observed subtle gray matter density reductions in left middle occipital, left superior parietal bilateral frontal lobe. When applying a more liberal threshold of *p* < 0.001 (uncorrected for multiple comparisons), correlations between entorhinal [^18^F]flortaucipir BP_ND_ and gray matter density reductions were observed in the medial, inferior, and lateral temporal lobes. Correlations between higher Braak III–IV [^18^F]flortaucipir BP_ND_ and smaller gray matter density comprised large parts of the lateral and inferior temporal cortices. In addition, higher [^18^F]flortaucipir BP_ND_ was correlated to lower GM density in the precuneus, superior frontal, left superior temporal, and right inferior and middle occipital lobes. When compared to the gray matter density pattern related to [^18^F]flortaucipir BP_ND_ in Braak III–IV, [^18^F]flortaucipir BP_ND_ in Braak V–VI was associated with slightly less widespread gray matter density reduction in the temporal cortices, but additionally included diminished gray matter in the bilateral inferior, middle and superior occipital, left superior parietal, and bilateral superior frontal lobes. Repeating the analyses using non-partial-volume-corrected BP_ND_ images yielded similar atrophy patterns (Additional file 4: Figure S4). Sensitivity analyses revealed that for the total sample and when stratified by clinical stage, no associations between hippocampal [^18^F]flortaucipir BP_ND_ and GM density were present (Additional file 8: Figure S5).

## Discussion

Using quantitative analysis of specific tau binding, we found that higher tau load in entorhinal, limbic, and neocortical regions was strongly correlated with local and distant cortical smaller gray matter density, which was largely attributable to the patients with MCI or AD dementia. By contrast, higher specific [^18^F]flortaucipir binding was not associated with greater atrophy in cognitively unimpaired subjects. [^18^F]Flortaucipir in the entorhinal cortex, but not in the hippocampus, was related to atrophy in the medial temporal lobe (MTL) structures. Tau load in cortical regions beyond the MTL was associated with atrophy patterns that encompassed widespread cortical regions, such as the inferior temporal, precuneus, occipital, and frontal lobes, but did not include the medial temporal lobe structures.

We observed widespread associations between tau and atrophy across a sample of subjects with SCD, MCI and AD, which is in line with neuropathology and imaging studies [10, 11, 16, 25, 43]. Amongst amyloid-β positive patients with MCI and AD dementia, there were two remarkable findings. First, [^18^F]flortaucipir BP_ND_ in entorhinal cortex was most strongly associated with medial temporal lobe gray matter density reduction, while [^18^F]flortaucipir in later AD-associated regions was related to atrophy in widespread regions, except for the medial temporal lobe. The voxelwise atrophy pattern associated with tau in Braak III–IV mainly comprised lateral and inferior temporal lobes, indicating a local association between tau and atrophy. However, distant effects were also observed, as tau in Braak III–IV was associated with more widespread cortical atrophy including frontal, occipital and parietal regions. When studying local and distant effects across 9 regions of interest (Fig. 4), we observed both local and distant associations between tau pathology and gray matter density across all regions outside the medial temporal lobe. In fact, for most regions, local effect sizes were in the same range as distant effect sizes, suggesting that tau had an equal effect on local and distant gray matter atrophy. In contrast, atrophy in the MTL was only found for higher levels of entorhinal tau, suggesting a local effect of tau in these regions only. Previous studies in AD dementia mostly focused on local tau-related neurodegeneration. In a number of studies, local effects of [^18^F]flortaucipir on [^18^F] FDG hypometabolism across neocortical brain regions were shown [20, 23, 26]. In a study amongst 30 mild AD patients, local effects of [^18^F]flortaucipir on brain volumes were seen in occipital regions, while when assessing distant effects, tau was mainly associated with smaller gray matter density in adjacent areas, with the majority of effects found in frontal and occipital lobes [16]. In amyloid-positive cognitively normal and MCI subjects, both local and nonlocal effects of [^18^F]flortaucipir on medial lobe atrophy were observed [18].

Second, in sensitivity analyses, we observed that in MCI/AD, entorhinal tau, but not hippocampal tau, was associated with lower GM density. The absence of associations between tau in the hippocampus and atrophy could be due to our relatively young sample (mean age 65 ± 8 years) and the inclusion of subjects with non-amnestic presentations, which are associated with an atrophy pattern more pronounced in cortical regions and relative sparing of hippocampus and other medial temporal structures [44–46]. As a result, tau pathology in the hippocampus in this sample showed less variation and was possibly not a sensitive marker of underlying AD pathology. This is underscored by the fact that the hippocampus was the only ROI where we did not find differences in [^18^F]flortaucipir BP_ND_ between MCI/AD and controls. Alternatively, hippocampal [^18^F]flortaucipir signal was largely influenced by spill-in from off-target binding in the nearby entorhinal cortex. It has been suggested that spill-out from the choroid plexus could be reduced using PVC methods [12, 24, 47]. In a previous study performed by our group, by using eroded and PVC data, correlations between the choroid plexus and hippocampus diminished, suggesting that spill-in in the hippocampus was reduced [47]. In the current dataset however, no associations between hippocampal [^18^F]flortaucipir BP_ND_ and atrophy were observed, even when using PVE-corrected data.

The extent of tau pathology in more advanced Braak stages (i.e., Braak V–VI ROI or parietal, frontal, and occipital ROIs) was not related to greater atrophy than tau in regions corresponding to Braak stage III–IV (i.e., medial temporal ROI). This could indicate that the strength of the relationship between tau in Braak V–VI and atrophy reduced due to the inclusion of subjects with non-amnestic phenotypes of AD, as strong focal associations have been shown between tau PET and neurodegeneration in AD clinical variants [25, 26]. As a result of the size of Braak V–VI (which encapsulated almost the entire neocortex), these local associations between tau and gray matter could be masked due to dilution of [^18^F]flortaucipir BP_ND_ in such a large ROI. This is underscored by the strong associations between occipital and frontal [^18^F]flortaucipir BP_ND_ and gray matter density reduction. Alternatively, the presence of atrophy in the more severely affected patients, assumingly those with the highest tau deposition in ROI Braak V–VI, induced more partial volume effects (PVE), thereby artificially lowering [^18^F]flortaucipir BP_ND_ in ROI Braak V–VI and thus diminishing correlations with gray matter. To overcome this problem, we used a validated PVC method [41] to reduce PVE. Although we did not observe differences in analyses with and without PVE correction, the influence of PVE cannot be completely ruled out.

We did not observe a relationship between [^18^F]flortaucipir tau PET and atrophy in cognitively unimpaired individuals. In our sample, these subjects showed fairly low BP_ND_ values across all regions, hampering the sensitivity to detect associations between tracer uptake and atrophy. Furthermore, since expected gray matter density loss in preclinical AD is presumably subtle, AD-related effects were possibly not detected due to pre-morbid differences in brain volume between subjects and age-related atrophy. It could be argued that the presence of tau pathology in the medial temporal lobe is not a sensitive marker of disease severity, but is partially related to chronological aging [48]. The term primary age-related tauopathy (PART) [49] was coined in neuropathological studies to describe the presence of neurofibrillary tangles in Braak stage I–IV in the absence of amyloid-β pathology and cognitive impairment. Although some argue that PART represents an early stage of the AD continuum, where Aβ pathology will appear inevitably with progression of the disease process [50], others propose that PART is a non-AD entity, largely associated with age [49]. An integrative view would be that amyloid-β and PART arise independently, but that their co-occurrence induces spreading of tau outside the medial temporal lobe. This latter process is then associated with downstream neurodegenerative processes and cognitive decline [51–53]. If the presence of tau in cognitively normal subjects is due to chronological aging (e.g., PART), while associations between tau and neurodegeneration in the AD clinical spectrum are evident, amongst cognitively normal individuals, the presence of tau might not be closely related with more downstream neurodegenerative processes. However, relationships between tau PET and neurodegeneration in cognitively unimpaired subjects have been reported. A relatively large study showed a negative association between local [^18^F]flortaucipir and cortical thickness in the entorhinal, fusiform, inferior, and middle temporal cortices and temporal poles [17]. The presence of amyloid-β possibly has an important role on the relationship between tau and neurodegeneration. This is supported by two [^18^F]FDG-PET studies. In the first study, the local association between [^18^F]flortaucipir and lower glucose metabolism in entorhinal and inferior temporal cortices increased with increasing amyloid-β load amongst cognitively normal participants. While entorhinal [^18^F]flortaucipir was directly associated with distant hypometabolism in temporal regions, inferior temporal [^18^F]flortaucipir was only associated with medial and lateral temporal hypometabolism amongst amyloid-β-positive subjects [21]. In the second study, tau was associated with an increase in glucose metabolism in amyloid-β negative subjects, while in amyloid-β-positive subjects tau in the medial temporal lobe was associated with FDG hypometabolism and tau outside the medial temporal lobe with both increased and decreased metabolism [22].

The main strength of this study is the memory clinic-based cohort that covered the spectrum from cognitively normal to AD dementia. As opposed to other studies, we included both cognitively impaired and unimpaired individuals, and stratified analyses to provide results for each group. We quantified tau load using full dynamic scans and the previously validated simplified reference method RPM [38]. In contrast to the more widely used method SUVr, the advantage of this method is that it takes into account tracer delivery and wash-out and as a result measures specific binding of the tracer [^18^F]flortaucipir to tau pathology [37]. Furthermore, we assessed both local and distant associations between tau and atrophy. A potential limitation of this study is that relationships between tau and atrophy could be influenced by amyloid-β. Due to a small sample size in controls and a variety of amyloid-β measures in MCI/AD (different PET tracers and CSF), we were not able to assess the effect of amyloid-β on this relationship. One could argue that with the use of pre-defined regions of interest for tau pathology, variations in the in vivo nature of the tau PET signal was lost. However, multiple PET studies consistently reported the spread of tau in Braak regions, and this approach allows comparing current results to neuropathological data. Furthermore, analyzing tau PET signal with a more unbiased approach, using ROIs comprising major brain lobes, yielded comparable results. As an effect of the low spatial resolution of PET, measures in small regions could be less robust than measures in larger areas. Tau PET signal in subjects with extensive atrophy could be influenced by partial volume effects. As a result, tau measures in more advanced Braak regions could be less reliable. All analyses were performed in template space. The transformation from native space to template space could have induced noise, which could have influenced the sensitivity of our analyses. Due to the cross-sectional nature of this study, a temporal order of tau deposition and neurodegeneration could not be established. Since the exact mechanisms underlying tau-induced neurodegeneration are still unknown, it could be possible that the deposition of tau neurofibrillary tangles and neurodegeneration occur in parallel. Moreover, it is under debate if hyperphosphorylated tau aggregates are necessary for neurodegeneration or that toxic monomeric or oligomeric tau species cause the deterioration and loss of neuronal function [6].

## Conclusions

In summary, our findings indicate that particularly amongst MCI/AD patients, [^18^F]flortaucipir in the entorhinal, limbic, and neocortical regions was related to cortical atrophy. While entorhinal tau was closely related to local atrophy, tau in the limbic and neocortical regions was associated with atrophy in both the local and distant regions. We observed no effects of tau on smaller gray matter density amongst cognitively normal controls. Our present study highlights that in clinically affected AD patients, the presence and spatial distribution of phosphorylated tau is tightly linked to neurodegeneration.

## Additional files

Additional file 1: **Figure S1**. Correlations between regional [^18^F]flortaucipir BP_ND_ and GM density in controls (PNG 2070 kb)

Additional file 2: **Figure S2**. Correlations between [^18^F]flortaucipir BP_ND_ and voxelwise GM density in controls. Displayed are the results of voxelwise regression analyses with [^18^F]flortaucipir BP_ND_ per Braak ROI (top) as predictor and GM density as dependent variables. Analyses are adjusted for age, sex, TIV and PET scanner type. [^18^F]Flortaucipir BP_ND_ images are partial volume corrected. *p* values are set at 0.001, uncorrected for multiple comparisons. (PNG 2523 kb)

Additional file 3: **Figure S3**. Correlations between [^18^F]flortaucipir BP_ND_ and voxelwise GM density in MCI/AD. Displayed are the results of voxelwise regression analyses with [^18^F]flortaucipir BP_ND_ per Braak ROI (top) as predictor and GM density as dependent variables. Analyses are adjusted for age, sex, TIV, and PET scanner type. [^18^F]Flortaucipir BP_ND_ images are partial volume corrected. *p* values are set at 0.05, family-wise error corrected. (PNG 2529 kb)

Additional file 4: **Figure S4**. Correlations between [^18^F]flortaucipir BP_ND_ in and voxelwise GM density in MCI/AD for data without partial volume correction. Displayed are the results of voxelwise regression analyses with [^18^F]flortaucipir BP_ND_ per Braak ROI (top) as predictor and GM density as dependent variables. Analyses are adjusted for age, sex, TIV, and PET scanner type. [^18^F]Flortaucipir BP_ND_ images are partial volume corrected. *p* values are set at 0.05, family-wise error corrected. *Remake of Additional file 2: Figure S2, but now contains data without partial volume correction (PNG 1804 kb)

Additional file 5: **Table S1**. Coordinates of local maxima (*p* = 0.05, FWE) (DOCX 13 kb)

Additional file 6: **Table S2**. Correlations between [^18^F]flortaucipir in lobar ROIs and GM density. Displayed are standardized betas. Analyses are adjusted for age, sex, TIV, and PET scanner type. *p < 0.05, **p < 0.01, ***p < 0.001, #FDR corrected (DOCX 16 kb)

Additional file 7: **Table S3**. Correlations between [^18^F]flortaucipir in lobar ROIs and GM density for non-PVE-corrected [^18^F]flortaucipir images Displayed are standardized betas. Analyses are adjusted for age, sex, TIV, and PET scanner type. (DOCX 15 kb)

Additional file 8: **Figure S5**. (PNG 1.7 kb)

## Data Availability

The datasets analyzed during the current study may be available from the corresponding author on reasonable request.

## Abbreviations

AAL: Automatic anatomic labeling
AD: Alzheimer’s disease
Aβ: Amyloid-beta
BP_ND_: Non-displaceable binding potential
CSF: Cerebrospinal fluid
CT: Computed tomography
FDG: Fluorodeoxyglucose
FDR: False discovery rate
GM: Gray matter
MCI: Mild cognitive impairment
MNI: Montreal Neurological Institute
MRI: Magnetic resonance imaging
PART: Primary age-related tauopathy
PET: Positron emission tomography
PiB: Pittsburg Compound B
PVC: Partial volume correction
PVE: Partial volume effect
ROI: Region of interest
RPM: Receptor parametric mapping
SCD: Subjective cognitive decline
TIV: Total intracranial volume
