# Supplementary figures and images for: Associations between quantitative [18F]flortaucipir tau PET and atrophy across the Alzheimer’s disease spectrum
Source: Alzheimers Res Ther. 2019 Jul 4;11:60. doi: 10.1186/s13195-019-0510-3 (PMC6610969; doi:10.1186/s13195-019-0510-3)

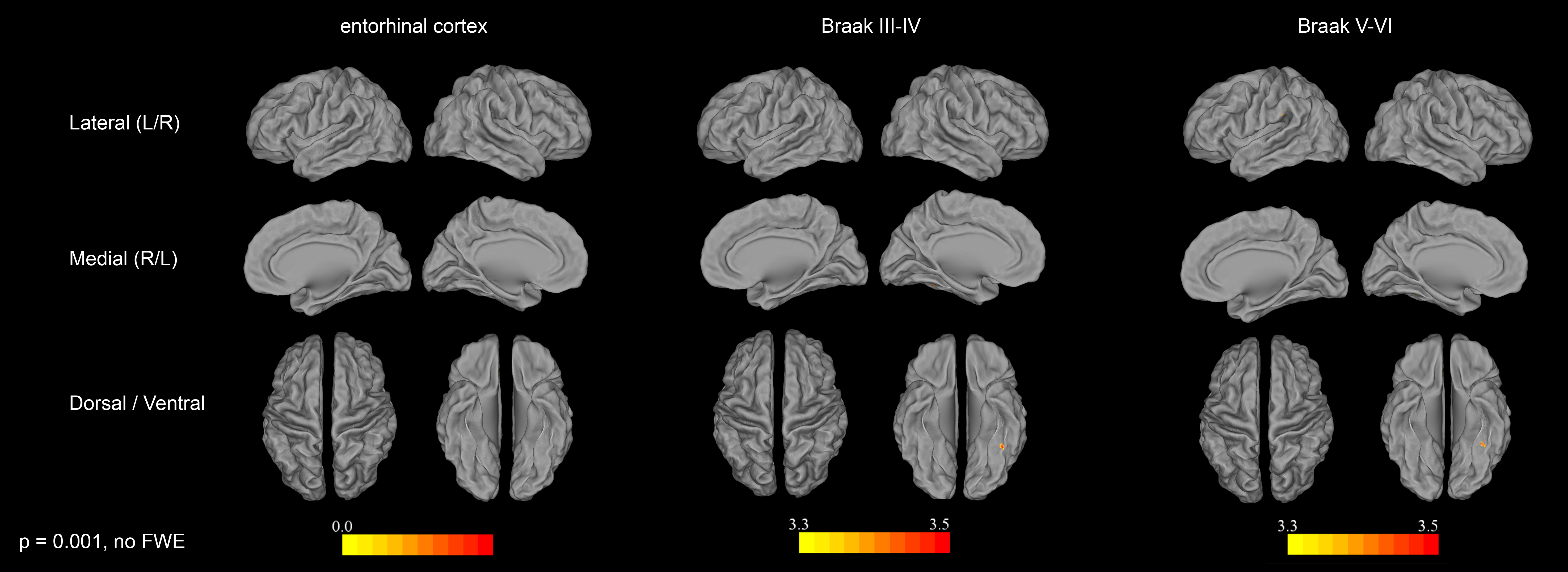

Supplement: Supplementary file 1 — Figure S1. Correlations between regional [18F]flortaucipir BPND and GM density in controls (PNG 2070 kb) [file 13195_2019_510_MOESM1_ESM.png]

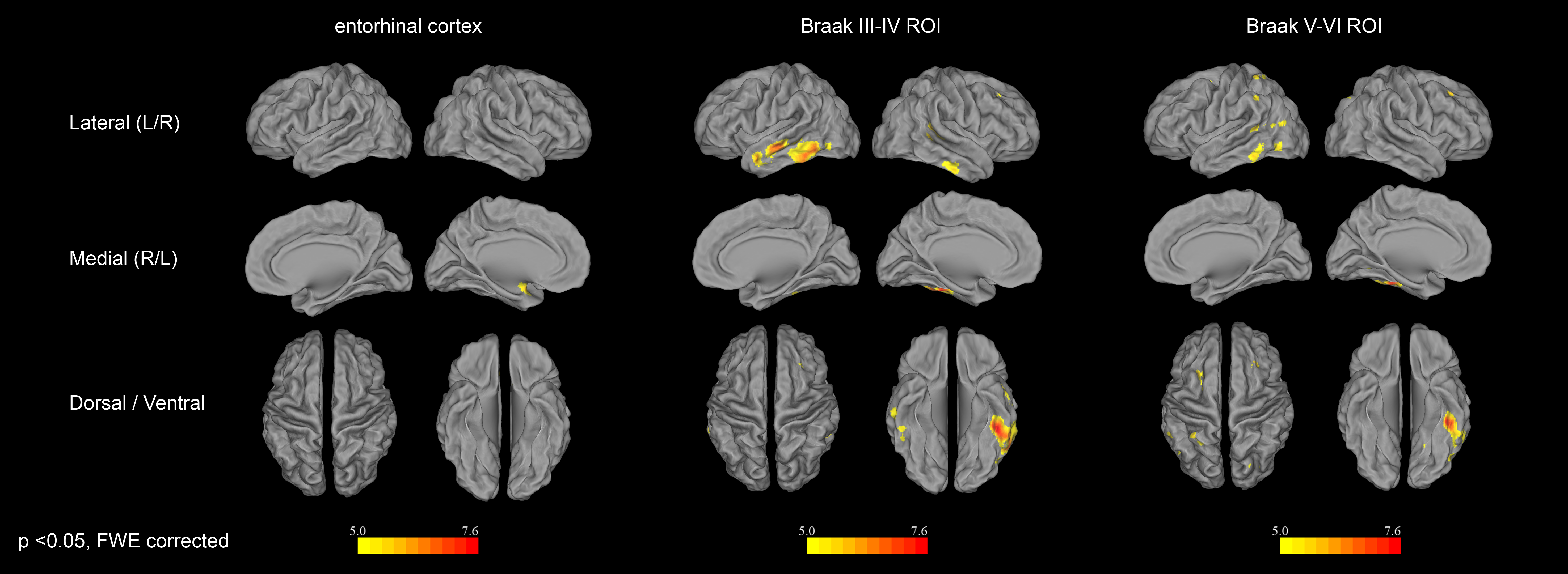

Supplement: Supplementary file 2 — Figure S2. Correlations between [18F]flortaucipir BPND and voxelwise GM density in controls. Displayed are the results of voxelwise regression analyses with [18F]flortaucipir BPND per Braak ROI (top) as predictor and GM density as dependent variables. Analyses are adjusted for age, sex, TIV and PET scanner type. [18F]Flortaucipir BPND images are partial volume corrected. p values are set at 0.001, uncorrected for multiple comparisons. (PNG 2523 kb) [file 13195_2019_510_MOESM2_ESM.png]

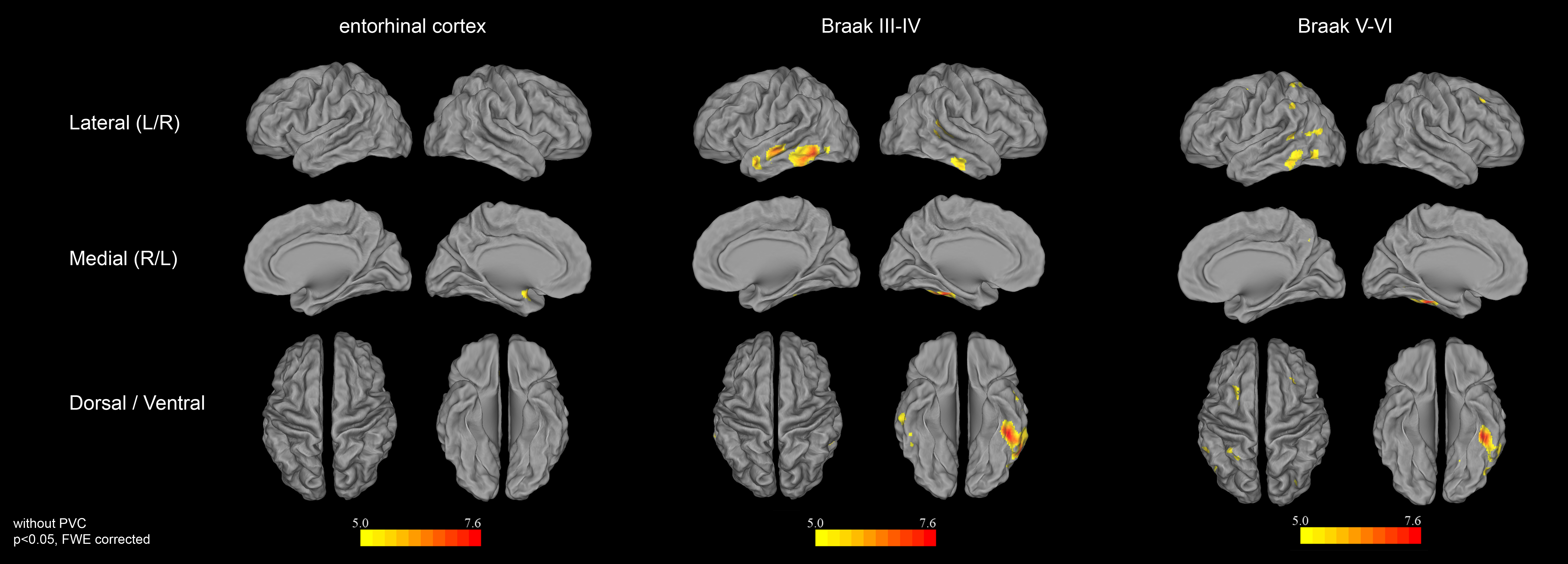

Supplement: Supplementary file 3 — Figure S3. Correlations between [18F]flortaucipir BPND and voxelwise GM density in MCI/AD. Displayed are the results of voxelwise regression analyses with [18F]flortaucipir BPND per Braak ROI (top) as predictor and GM density as dependent variables. Analyses are adjusted for age, sex, TIV, and PET scanner type. [18F]Flortaucipir BPND images are partial volume corrected. p values are set at 0.05, family-wise error corrected. (PNG 2529 kb) [file 13195_2019_510_MOESM3_ESM.png]

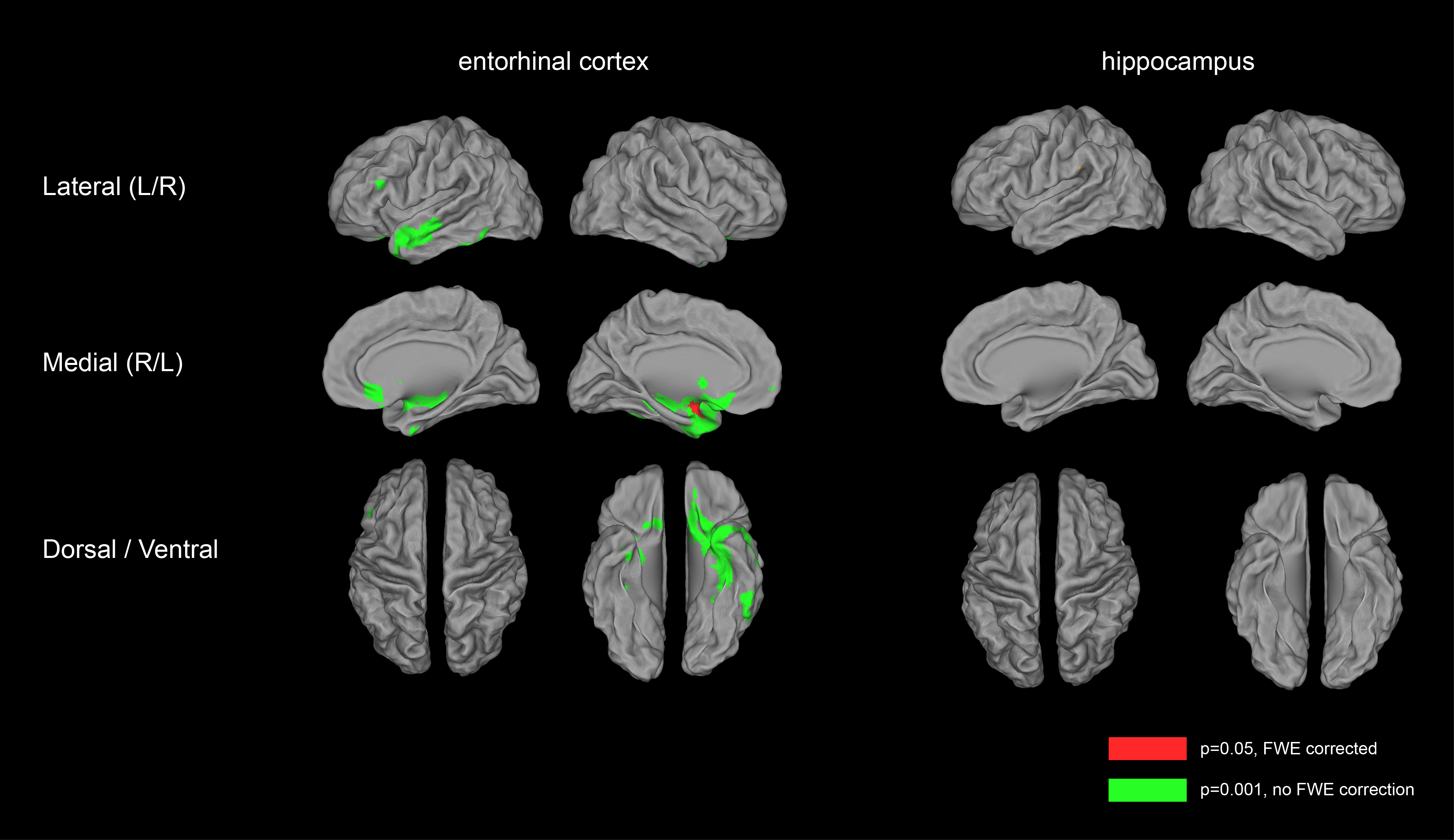

Supplement: Supplementary file 4 — Figure S4. Correlations between [18F]flortaucipir BPND in and voxelwise GM density in MCI/AD for data without partial volume correction. Displayed are the results of voxelwise regression analyses with [18F]flortaucipir BPND per Braak ROI (top) as predictor and GM density as dependent variables. Analyses are adjusted for age, sex, TIV, and PET scanner type. [18F]Flortaucipir BPND images are partial volume corrected. p values are set at 0.05, family-wise error corrected. *Remake of Additional file 2: Figure S2, but now contains data without partial volume correction (PNG 1804 kb) [file 13195_2019_510_MOESM4_ESM.png]
