# Supplementary material for: Associations between quantitative [18F]flortaucipir tau PET and atrophy across the Alzheimer’s disease spectrum
Source: Alzheimers Res Ther. 2019 Jul 4;11:60. doi: 10.1186/s13195-019-0510-3 (PMC6610969; doi:10.1186/s13195-019-0510-3)
Supplement: Supplementary file 5 — Table S1. Coordinates of local maxima (p = 0.05, FWE) (DOCX 13 kb) [file 13195_2019_510_MOESM5_ESM.docx]

***Table S1: Coordinates of local maxima (p=0.05, FWE)***

| **Coordinates of local maxima** | | | **Anatomical label** | **T-value** | **p** | **Cluster size** |
| --- | --- | --- | --- | --- | --- | --- |
| *x* | *y* | *z* |  |  |  |  |
| **MCI/AD entorhinal cortex** | | | | | | |
| -29 | -2 | -18 | Amygdala L | 5.07 | 0.000 | 43 |
| **MCI/AD Braak III-IV** | | | | | | |
| -53 | -56 | -11 | Temporal Inf L | 7.84 | 0.000 | 5106 |
| 53 | -44 | 12 | Temporal Mid R | 6.39 | 0.001 | 557 |
| 56 | -21 | -27 | Temporal Inf R | 5.89 | 0.004 | 368 |
| 48 | -48 | -24 | Temporal Inf R | 5.39 | 0.017 | 48 |
| 35 | -78 | 27 | Occipital Mid R | 5.31 | 0.022 | 22 |
| 26 | 21 | 44 | Frontal Sup R | 5.14 | 0.037 | 14 |
| 50 | -36 | -23 | Temporal Inf R | 5.13 | 0.038 | 10 |
| **MCI/AD Braak V-VI** | | | | | | |
| -41 | -36 | -21 | Fusiform L | 7.50 | 0.000 | 2115 |
| -41 | -74 | 12 | Occipital Mid L | 5.94 | 0.003 | 347 |
| -35 | -45 | 63 | Parietal Sup L | 5.88 | 0.004 | 127 |
| -26 | 11 | 51 | Frontal Mid L | 5.88 | 0.004 | 89 |
| -20 | -63 | -6 | Lingual L | 5.81 | 0.005 | 32 |
| -54 | -50 | 39 | Parietal Inf L | 5.78 | 0.005 | 78 |
| 27 | 21 | 45 | Frontal Mid R | 5.77 | 0.005 | 135 |
| -20 | 2 | 53 | Frontal Sup L | 5.40 | 0.017 | 10 |
| 14 | -68 | 57 | Parietal Sup R | 5.39 | 0.018 | 15 |
| 33 | -78 | 27 | Occipital Mid R | 5.38 | 0.018 | 47 |
| 0 | -62 | 41 | Precuneus L | 5.38 | 0.018 | 17 |
| -57 | -41 | -8 | Temporal Mid L | 5.26 | 0.026 | 11 |
| -32 | -92 | 9 | Occipital Mid L | 5.24 | 0.028 | 14 |
| 54 | -45 | 17 | Temporal Sup R | 5.16 | 0.035 | 10 |
