# Supplementary material for: Associations between quantitative [18F]flortaucipir tau PET and atrophy across the Alzheimer’s disease spectrum
Source: Alzheimers Res Ther. 2019 Jul 4;11:60. doi: 10.1186/s13195-019-0510-3 (PMC6610969; doi:10.1186/s13195-019-0510-3)
Supplement: Supplementary file 6 — Table S2. Correlations between [18F]flortaucipir in lobar ROIs and GM density. Displayed are standardized betas. Analyses are adjusted for age, sex, TIV, and PET scanner type. *p < 0.05, **p < 0.01, ***p < 0.001, #FDR corrected (DOCX 16 kb) [file 13195_2019_510_MOESM6_ESM.docx]

**Table S2: Correlations between [18F]Flortaucipir in lobar ROIs and GM volumes.** Displayed are standardized betas. Analyses are adjusted for age, sex, TIV and PET scanner type. *p<0.05, **p<0.01, ***p<0.001, #FDR corrected

|  | Entorhinal | Hippocampus | MTL | LTL | MPL | LPL | Occipital | Frontal | Global |
| --- | --- | --- | --- | --- | --- | --- | --- | --- | --- |
| TOTAL | | | | | | | | | |
| Entorhinal | **-0.42***#** | 0.10 | **-0.31***#** | **-0.39***#** | **-0.31**#** | **-0.32***#** | **-0.31***#** | **-0.30**#** | **-0.37***#** |
| Hippocampus | **-0.51***#** | 0.09 | **-0.38***#** | **-0.41***#** | **-0.38***#** | **-0.39***#** | **-0.36***#** | **-0.35***#** | **-0.41***#** |
| MTL | **-0.49***#** | 0.08 | **-0.37***#** | **-0.41***#** | **-0.37***#** | **-0.38***#** | **-0.35***#** | **-0.34***#** | **-0.41***#** |
| LTL | **-0.42***#** | 0.04 | **-0.30***#** | **-0.59***#** | **-0.54***#** | **-0.57***#** | **-0.49***#** | **-0.55***#** | **-0.60***#** |
| MPL | **-0.36***#** | 0.03 | **-0.28***#** | **-0.48***#** | **-0.52***#** | **-0.53***#** | **-0.51***#** | **-0.51***#** | **-0.55***#** |
| LPL | **-0.36***#** | 0.03 | **-0.28***#** | **-0.45***#** | **-0.54***#** | **-0.54***#** | **-0.49***#** | **-0.51***#** | **-0.54***#** |
| Occipital | **-0.392***#** | 0.06 | **-0.28***#** | **-0.53***#** | **-0.63***#** | **-0.63***#** | **-0.64***#** | **-0.52***#** | **-0.62***#** |
| Frontal | **-0.335***#** | 0.04 | **-0.24**#** | **-0.35***#** | **-0.35***#** | **-0.37***#** | **-0.30***#** | **-0.43***#** | **-0.41***#** |
| Global | **-0.377***#** | 0.06 | **-0.27***#** | **-0.45***#** | **-0.46***#** | **-0.48***#** | **-0.42***#** | **-0.48***#** | **-0.50***#** |
| controls | | | | | | | | | |
| Entorhinal | 0.00 | 0.09 | 0.06 | -0.11 | -0.10 | -0.06 | 0.02 | -0.02 | -0.07 |
| Hippocampus | -0.09 | 0.14 | 0.03 | -0.06 | -0.12 | -0.07 | -0.02 | -0.03 | -0.06 |
| MTL | -0.08 | 0.12 | 0.02 | -0.09 | -0.13 | -0.09 | -0.02 | -0.04 | -0.08 |
| LTL | -0.09 | 0.08 | -0.01 | -0.14 | -0.12 | -0.16 | -0.03 | -0.15 | -0.14 |
| MPL | -0.12 | 0.02 | -0.08 | -0.20 | -0.18 | -0.19 | -0.17 | -0.22 | -0.23 |
| LPL | -0.03 | 0.11 | 0.04 | -0.04 | -0.11 | -0.08 | 0.00 | -0.22 | -0.10 |
| Occipital | -0.13 | -0.02 | -0.08 | -0.03 | -0.04 | -0.03 | 0.04 | -0.16 | -0.07 |
| Frontal | -0.20 | 0.02 | -0.07 | -0.09 | -0.22 | -0.17 | -0.07 | -0.12 | -0.13 |
| Global | -0.11 | 0.06 | -0.02 | -0.09 | -0.13 | -0.12 | -0.01 | -0.12 | -0.10 |
| MCI/AD | | | | | | | | | |
| Entorhinal | **-0.38***#** | 0.13 | -0.24 | -0.21 | -0.04 | -0.09 | -0.12 | -0.09 | -0.17 |
| Hippocampus | **-0.42***#** | 0.17 | **-0.27*** | -0.14 | -0.08 | -0.11 | -0.11 | -0.08 | -0.15 |
| MTL | **-0.40***#** | 0.15 | **-0.25*** | -0.16 | -0.07 | -0.10 | -0.11 | -0.09 | -0.16 |
| LTL | **-0.23*** | 0.14 | -0.06 | **-0.54***#** | **-0.43***#** | **-0.48***#** | **-0.39***#** | **-0.46***#** | **-0.56***#** |
| MPL | -0.12 | 0.18 | -0.01 | **-0.31**#** | **-0.41**#** | **-0.41***#** | **-0.40***#** | **-0.40***#** | **-0.44***#** |
| LPL | -0.13 | 0.15 | -0.05 | **-0.28*#** | **-0.43**#** | **-0.45***#** | **-0.39***#** | **-0.40***#** | **-0.45***#** |
| Occipital | -0.12 | **0.24*** | 0.05 | **-0.40***#** | **-0.56***#** | **-0.55***#** | **-0.59***#** | **-0.36**#** | **-0.54***#** |
| Frontal | **-0.21*** | 0.14 | -0.09 | **-0.25*** | -0.20 | **-0.26*** | -0.17 | **-0.38***#** | **-0.34**#** |
| Global | **-0.21*** | 0.18 | -0.06 | **-0.34***#** | **-0.34**#** | **-0.39***#** | **-0.32**#** | **-0.39***#** | **-0.44***#** |
