# Supplementary material for: Associations between quantitative [18F]flortaucipir tau PET and atrophy across the Alzheimer’s disease spectrum
Source: Alzheimers Res Ther. 2019 Jul 4;11:60. doi: 10.1186/s13195-019-0510-3 (PMC6610969; doi:10.1186/s13195-019-0510-3)
Supplement: Supplementary file 7 — Table S3. Correlations between [18F]flortaucipir in lobar ROIs and GM density for non-PVE-corrected [18F]flortaucipir images Displayed are standardized betas. Analyses are adjusted for age, sex, TIV, and PET scanner type. (DOCX 15 kb) [file 13195_2019_510_MOESM7_ESM.docx]

**Table S3: Correlations between [^18^F]Flortaucipir in lobar ROIs and GM volumes for non-PVE corrected [^18^F]Flortaucipir images** Displayed are standardized betas. Analyses are adjusted for age, sex, TIV and PET scanner type.

|  | Entorhinal | Hippocampus | MTL | LTL | MPL | LPL | Occipital | Frontal | Global |
| --- | --- | --- | --- | --- | --- | --- | --- | --- | --- |
| TOTAL | | | | | | | | | |
| Entorhinal | **-0.42** | -0.04 | **-0.33** | **-0.39** | **-0.30** | **-0.31** | **-0.30** | **-0.29** | **-0.36** |
| Hippocampus | **-0.50** | -0.05 | **-0.39** | **-0.41** | **-0.38** | **-0.38** | **-0.35** | **-0.34** | **-0.41** |
| MTL | **-0.49** | -0.05 | **-0.38** | **-0.41** | **-0.36** | **-0.37** | **-0.34** | **-0.34** | **-0.40** |
| LTL | **-0.43** | -0.05 | **-0.33** | **-0.59** | **-0.54** | **-0.57** | **-0.49** | **-0.55** | **-0.60** |
| MPL | **-0.37** | -0.07 | **-0.31** | **-0.48** | **-0.53** | **-0.53** | **-0.50** | **-0.51** | **-0.55** |
| LPL | **-0.37** | -0.08 | **-0.31** | **-0.44** | **-0.54** | **-0.54** | **-0.49** | **-0.51** | **-0.54** |
| Occipital | **-0.40** | -0.07 | **-0.32** | **-0.54** | **-0.63** | **-0.63** | **-0.64** | **-0.53** | **-0.63** |
| Frontal | **-0.33** | -0.05 | **-0.25** | **-0.35** | **-0.34** | **-0.36** | **-0.29** | **-0.43** | **-0.41** |
| Global | **-0.38** | -0.04 | **-0.29** | **-0.45** | **-0.46** | **-0.48** | **-0.42** | **-0.48** | **-0.50** |
| controls | | | | | | | | | |
| Entorhinal | -0.01 | 0.08 | 0.04 | -0.11 | -0.11 | -0.06 | 0.05 | 0.02 | -0.07 |
| Hippocampus | -0.10 | 0.11 | 0.01 | -0.07 | -0.13 | -0.07 | -0.01 | -0.01 | -0.07 |
| MTL | -0.09 | 0.09 | 0.00 | -0.09 | -0.14 | -0.09 | -0.00 | -0.02 | -0.08 |
| LTL | -0.13 | 0.06 | -0.03 | -0.15 | -0.14 | -0.16 | -0.03 | -0.11 | -0.14 |
| MPL | -0.15 | -0.02 | -0.10 | -0.21 | -0.20 | -0.20 | -0.16 | -0.22 | -0.24 |
| LPL | -0.06 | 0.08 | 0.01 | -0.05 | -0.12 | -0.08 | -0.01 | -0.20 | -0.11 |
| Occipital | -0.15 | -0.06 | -0.10 | -0.03 | -0.05 | -0.04 | 0.05 | -0.15 | -0.07 |
| Frontal | -0.21 | -0.02 | -0.10 | -0.10 | -0.23 | -0.18 | -0.06 | -0.09 | -0.14 |
| Global | -0.14 | 0.03 | -0.04 | -0.09 | -0.15 | -0.12 | -0.00 | -0.10 | -0.11 |
| MCI/AD | | | | | | | | | |
| Entorhinal | **-0.35** | 0.07 | -0.23 | -0.20 | -0.03 | -0.07 | -0.11 | -0.07 | -0.16 |
| Hippocampus | **-0.38** | 0.08 | **-0.26** | -0.13 | -0.07 | -0.08 | -0.10 | -0.06 | -0.13 |
| MTL | **-0.37** | 0.08 | **-0.24** | -0.15 | -0.05 | -0.08 | -0.10 | -0.07 | -0.14 |
| LTL | -0.20 | 0.13 | -0.06 | **-0.54** | **-0.43** | **-0.47** | **-0.38** | **-0.46** | **-0.55** |
| MPL | -0.11 | 0.14 | -0.02 | **-0.30** | **-0.41** | **-0.41** | **-0.40** | **-0.41** | **-0.45** |
| LPL | -0.13 | 0.09 | -0.06 | **-0.28** | **-0.42** | **-0.44** | **-0.39** | **-0.41** | **-0.45** |
| Occipital | -0.12 | 0.19 | 0.01 | **-0.41** | **-0.56** | **-0.56** | **-0.60** | **-0.37** | **-0.55** |
| Frontal | -0.18 | 0.09 | -0.08 | **-0.24** | -0.19 | -0.25 | -0.16 | **-0.38** | **-0.34** |
| Global | -0.19 | 0.14 | -0.07 | **-0.34** | **-0.34** | **-0.38** | **-0.32** | **-0.39** | **-0.44** |
